# Symptomatic Therapy and Rehabilitation in Primary Progressive Multiple Sclerosis

**DOI:** 10.1155/2011/740505

**Published:** 2011-10-17

**Authors:** Fary Khan, Bhasker Amatya, Lynne Turner-Stokes

**Affiliations:** ^1^Department of Medicine, Dentistry and Health Sciences at The University of Melbourne, Royal Melbourne Hospital and Western Health, Rehabilitation Service—Royal Melbourne Hospital, Poplar Road, Parkville, Melbourne, VIC 3052, Australia; ^2^Department of Rehabilitation Medicine, Royal Melbourne Hospital, 34-54 Poplar Road Parkville, Melbourne, VIC 3052, Australia; ^3^Regional Rehabilitation Unit, Northwick Park Hospital, Watford Road, Harrow, Middlesex HA1 3UJ, UK

## Abstract

Multiple sclerosis (MS) is an autoimmune inflammatory demyelinating disease of the central nervous system and a major cause of chronic neurological disability in young adults. Primary progressive MS (PPMS) constitutes about 10% of cases, and is characterized by a steady decline in function with no acute attacks. The rate of deterioration from disease onset is more rapid than relapsing remitting and secondary progressive MS types. Multiple system involvement at onset and rapid early progression have a worse prognosis. PPMS can cause significant disability and impact on quality of life. Recent studies are biased in favour of relapsing remitting patients as treatment is now available for them and they are more likely to be seen at MS clinics. Since prognosis for PPMS is worse than other types of MS, the focus of rehabilitation is on managing disability and enhancing participation, and application of a “neuropalliative” approach as the disease progresses. This chapter presents the symptomatic treatment and rehabilitation for persons with MS, including PPMS. A multidisciplinary approach optimizes the intermediate and long-term medical, psychological and social outcomes in this population. Restoration and maintenance of functional independence and societal reintegration, and issues relating to quality of life are addressed in rehabilitation processes.

## 1. Background

Multiple sclerosis (MS) is a chronic inflammatory demyelinating disease of the central nervous system (CNS) affecting about 2.5 million persons' worldwide [1]. It is the commonest cause of chronic neurological disability in young adults. MS is complex and the exact pathogenesis is unclear. The various disease courses in persons with MS (pwMS) are shown in Box 1. One recent survey of 878 persons with primary progressive MS (PPMS) [2] were found to have a shorter median time to death from onset and a higher relative risk of dying despite the fact that persons with PPMS live for years with many disabilities that can cause limitation in function and restriction in participation and impact quality of life (QoL) [3].

The natural history of PPMS is less well known compared with other MS disease courses. Primary progressive MS occurs in approximately 10% of pwMS and is primarily progressive from onset. Approximately <5% of pwMS may present with a progressive course although these patients also experience occasional attacks, the progressive relapsing MS type (PRMS) (see Box 1). The diagnostic criteria for PPMS are shown in Table 1. It is thought to be different genetically from the relapsing remitting MS (RRMS) and in MRI behaviour from secondary progressive MS type (SPMS) [4, 5]. In PPMS, the paucity of MRI detectable disease activity is in contrast with the observed accumulation of irreversible disability [6]. Quantitative MRI studies [7] have highlighted the role of brain tissue damage outside T2 visible lesions in the pathophysiology of PPMS. Kutzelnigget al. [8] showed that diffuse white matter injury and cortical demyelination were hallmarks of progressive MS, occurring on a background of a global “low burning” inflammatory response with focal lesion load. The contribution of cord damage to the severity and evolution of PPMS has also been evident in several MRI studies [9, 10]. 

The overall female preponderance in MS is less applicable to PPMS. The rate of deterioration from disease onset in PPMS is more rapid, with higher relative mortality than other MS types [13]. Those with shorter time from onset to disability and those with involvement of three or more neurological symptoms at onset have a worse prognosis [14]. However prognosis in PPMS is not dependent on age, gender, or type of neurological system involvement at onset. A minority of people with PPMS can have a distinct relapse even decades after onset of progressive deterioration [14]. 

Recent nonpopulation-based studies are biased towards RRMS as treatment is now available for reducing the number of relapses and delaying disease progression, so they are more likely to be reviewed at MS clinics. All suggested drug treatments for PPMS are empiric as there is no convincing trial evidence of effectiveness for disease modifying therapy [17]. An individual with PPMS, therefore, has limited drug therapy options and may benefit from a symptomatic and supportive rehabilitation approach aimed at reducing symptoms and limitations at the level of activity and participation. 

**Figure 1:**
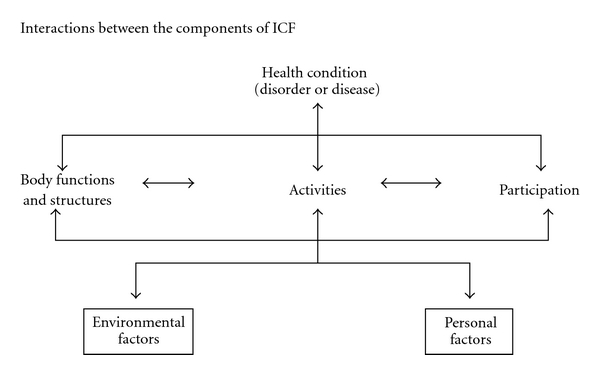
Components of international classification of functioning disability and health. (Printed with permission: WHO-ICF [15].)

Studies of pwMS have identified a range of impairments, limitations in activity (disability), and restriction in participation using the World Health Organization's International Classification of Functioning, Health, and Disability (ICF) Figure 1 [15, 18, 19]. These ICF domains have complex interactions and need evaluation and management through holistic interventions, which include personal and environmental factors. Recent MS expert consensus identified and recommended the “Core” set (minimum data set) of ICF categories for pwMS for comprehensive multidisciplinary assessments in the domains of body function, body structure, activities, and participation and environmental factors [18] (Tables 2–3). Much of the clinical focus has traditionally been on the physical aspects of MS but improved understanding of MS indicates involvement of multiple systems (cognition, memory, and emotional control). The combined effect of these impairments in a pwMS leads to greater disability than the sum of the individual impairments together. This may explain why some pwMS may not perform as well as expected [20].

## 2. Rehabilitation

Rehabilitation is defined as a problem solving educational process aimed at reducing disability and limitation in participation [21]. Rehabilitation interventions comprise expert MD assessments evaluated through appropriate outcome measures [22] using functional goal-oriented approaches such as clinical pathways [23] to target patient priorities [24]. Goal setting is an integral part of rehabilitation, as it encourages participants to set their own goals and priorities, and supports team communication and coordination. The key subcomponents and phases of the rehabilitation process [16] are shown in Boxes 2 and 3.

Existing clinical guidelines and frameworks [26, 27] for PPMS recommend comprehensive, flexible coordinated multidisciplinary (MD) care and appropriate followup, education, and support for patients and carers to address the limitations in activity and participation levels. Key issues for those severely affected include respite, community and/or long-term care, and community mobility. Early referral for rehabilitation enables strategies to restore recent functional deterioration [26]. In those severely affected, rehabilitation input can provide a modified environment and adaptive equipment to restore some functional independence. Caregiver education and support can reduce the burden of care. Rehabilitation can address QoL issues and direct care with other health professionals [28]. Importantly, “crisis management” should be avoided, planned management of disability and deficits should be anticipated (over time), and appropriate mechanisms that accommodate and facilitate functional independence provided. Some of the challenges to MS rehabilitation are shown in Box 4.

## 3. Evidence for MS Rehabilitation

A recent systematic review supports the effectiveness of MD rehabilitation programs in inpatient and ambulatory settings in terms of improvements in activity (disability) and participation [29]. Although there is evidence for some unidisciplinary rehabilitation interventions for pwMS, such as physical therapy [30], the evidence for others-occupational therapy [31] and psychological therapies [32] is less compelling. There is reasonable evidence to support cognitive behaviour approaches for depression in helping people adjust to, and cope with, having MS [33]. A recent randomized controlled trial showed the duration of benefit of rehabilitation in reducing disability persists for about 12 months [34] but not the positive effects on QoL and emotional well-being. Effects on QoL are often difficult to quantify in chronic conditions because of “response shift” or the change in internal values, or conceptualization of QoL, so that pwMS may reassess their perceived limitations of daily living and reset goals and consider the impact of their MS less marked than they thought formerly [35]. More studies are needed to assess impact of rehabilitation on QoL and to understand the response shift phenomenon in the MS population. 

Further, in addition to randomized controlled trial (RCT) methodology, a clinical practice improvement approach has also been applied to an inpatient MS cohort to understand the complex interplay of patient and process factors and impact on functional outcomes in rehabilitation for pwMS. In a pilot project (*n* = 24) [36], more than half of pwMS had moderate to severe fatigue, deficits in motor function and mood causing significant functional limitation, and two thirds required specialized nursing (e.g., continence care). Complexity of intervention was measured using the Northwick Park therapy dependency assessment (NPTDA) [37], which showed moderate dependency in physical, cognitive, and psychosocial domains. The NPTDA scores for these pwMS correlated strongly with FIM motor scores (Spearman rho −0.80) and Barthel index (rho −0.83) [36]. Further prospective studies are planned using appropriate tools to understand the “black box” of rehabilitation and the complex inter relationships of factors which impact function in this population. 

Khan et al. [38] examined outcomes following inpatient rehabilitation episodes (*n* = 1010) for pwMS using the Australian Rehabilitation Outcomes Centre Database. The majority of patients were female and following rehabilitation discharged to the community. Improvement in function was assessed using the functional independence measure (FIM), with subclasses of pwMS based on motor FIM scores for severity in functional limitation. Authors reported significant functional improvement (*P* = 0.001) with rehabilitation in most MS groups, with year to year trend towards reducing hospital length of stay and FIM efficiency although these did not reach significance.

## 4. Neuropalliative Approach in PPMS

**Figure 2:**
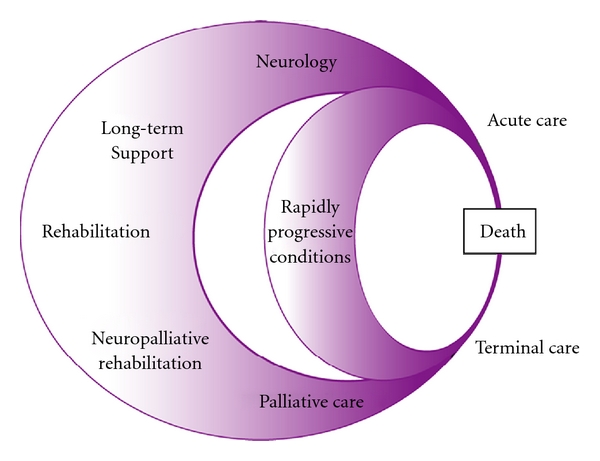
Life overlap diagram: interface between neurology, rehabilitation and palliative care in the management of persons with long-term neurological conditions and primary progressive multiple sclerosis. (Printed with permission: Turner-Stokes et al. [41].)

Since prognosis for PPMS is worse than other types of MS, the focus of rehabilitation is on managing disability and enhancing participation, and as disease progresses applying a “neuropalliative approach” [25]. The UK guidelines for managing long-term neurological conditions (LTNC) [39] are relevant to PPMS as they explore interaction between specialist neurology, rehabilitation, and palliative care services and how they work together to provide long-term support for people with LTNC and their carers. The key skills in neuropalliative care are shown in Table 4 [25]. Neurologists assess, diagnose, and manage disease, and the palliative care physicians' manage distressing symptoms (nausea, vomiting, and breathlessness), support and counsel the person and family, end-of-life issues, and provide advance care planning. Rehabilitation physicians contribute to care by managing disability and provide adaptive aids (mobility and communication), procedures for spasticity management (botulinum toxin and phenol), and pain and behaviour management. As disease progresses goal posts change and rehabilitation and palliative approaches can overlap, that is, “neuropalliation”. Many issues in PPMS can be managed by closer collaboration and cross referral between the above specialties [40, 41]. The life circles diagram Figure 2 [25] shows the overlap between roles of neurology, palliative care, and rehabilitation physicians which are relevant to people with PPMS.

## 5. Symptomatic Management and Rehabilitation

*This section outlines the symptomatic and disability management of persons with MS, including PPMS. As PPMS is a progressive disease, regular patient evaluation and reassessment of treatment and management is required. The rehabilitation intervention principles and “neuropalliation” apply to this patient population. The management strategy includes education, therapy input, and medications.*

PPMS presents primarily as progressive disease at onset; however, a minority may present with an acute relapse with a wide range of symptoms and signs. The most common patient reported symptoms that have a disabling impact are fatigue, mobility-related issues, and bladder and bowel dysfunction. One study of 101 pwMS showed the “linkage” of patient reported MS related problems and *degree of limitation* caused using the WHO ICF categories for the components: Body structure, Body Function, Activities and Participation, and Environmental factors [19], Table 5.

### 5.1. Disabilities in MS

#### 5.1.1. Fatigue

Fatigue is one of the most common symptoms in MS reported by up to 95% of pwMS. It is defined as “subjective” lack of physical or mental energy that is perceived by the individual or caregiver to interfere with usual activities and is present 60% of the time. In a sample of 656 patients with MS, 22% reported limitation in level of physical activity, 14% stated it required them to have more frequent rest breaks and 10% had to discontinue work due to fatigue [50]. Fatigue impacts on ability to work, social life and on activities of daily living. It is however difficult to predict and is unrelated to age, gender, disability as measured by Kurtzke Expanded Disability Status Scale (EDSS) [51] score or Neuro imaging status. Factors believed to contribute to fatigue in MS are summarized in Box 5. 

There are a number of measures of MS-related fatigue [48] (see Table 6).

Treatment of fatigue should be individualized based on the medical and functional status of each patient. The quality and quantity of fatigue, and its impact on function is obtained on history. Other non-MS causes should be excluded (anaemia and hypothyroidism) and contributing factors identified. Medications and side effects should be reviewed. Nonpharmacologic approaches include education for patient and family (avoid heat, use airconditioners, and cooling gel vests), address lifestyle factors, for example, diet and exercise, avoid physical activity at mid afternoon (as a small rise in core body temperature worsens fatigue and MS lassitude or lack of energy). Fatigue management and pacing (regular rest breaks between activities, i.e., pacing activities throughout the day), energy conservation, and work simplification strategies to decrease energy consumption and increase economy of effort (use of assistive devices, adaptive equipment such as long handled aids/grab rails, gait aids such as a walking frame, and ankle foot orthoses to improve gait efficiency) and improve overall fitness by structured exercise programs for aerobic capacity and endurance.

**Figure 3:**
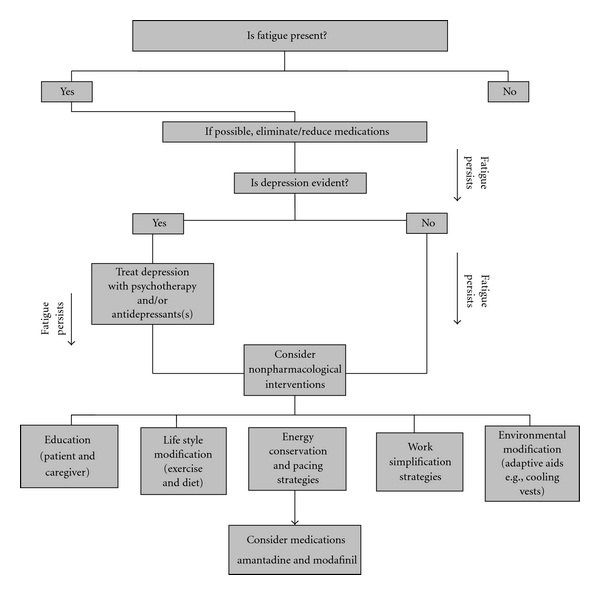
Clinical decision making flow chart for treating fatigue in MS. (Adapted from: MacAllister and Krupp [48].)

There is limited evidence supporting drug efficacy in MS related fatigue. Modafinil, a “wake promoting” agent that selectively works in the hypothalamic pathways, has been reported to improve fatigue in progressive MS. Amino pyridines (potassium channel blockers) and amantadine (N-methyl D-aspartate receptor antagonist) have been used, however systematic reviews failed to find evidence for efficacy or safety of their use [52]. Depression may contribute to fatigue in some cases and there is empiric support for use of antidepressants in MS related fatigue. A clinical decision making flowchart for managing fatigue in MS is shown in Figure 3 [48].

#### 5.1.2. Bladder, Bowel, and Sexual Dysfunction

Abnormalities in bladder function are primarily neurogenic and occur in over 80% of patients. Bladder dysfunction has a detrimental impact on mobility, everyday living activities, and on QoL in pwMS [53]. A recent RCT (*n* = 74) showed effectiveness of multidisciplinary rehabilitation for individualized bladder management program [54]. Approximately two thirds of patients on functional studies demonstrate overactive detrusor function, while the remainder exhibit underactivity. The external urethral sphincter complex may be synergic or dyssynergic with bladder contraction. Detrusor sphincter dyssynergia increases risk of pyelonephritis and renal failure due to combined effects of back pressure and reflux of infected urine into the kidney. Symptoms are unreliable in determining the precise underlying functional abnormality. Risk factors for progressive upper urinary tract dysfunction in MS, which require longer-term followup include: detrusor sphincter dyssynergia, age over 50 years, and male gender [55]. 

Urodynamic studies are mandatory to evaluate the pattern of neurogenic bladder dysfunction in an individual. On a day-to-day basis, general techniques for managing bladder care include scheduled fluid intake, timed voiding pattern, establishment of emptying techniques every three hours (tapping over suprapubic region, Credes maneuver), use of pads and undergarments, and use of bedside commode or urinal. Other simpler techniques such as voided volume charts will provide evidence of small frequent urine volumes suggesting detrusor hyperactivity. Postmicturition ultrasound should be performed regularly and if residual volumes exceed 100 mL, a regimen of intermittent clean self-catheterisation should be introduced. If this is not possible due to upper limb dysfunction or adductor spasm, long-term catheterisation should be considered. 

**Figure 4:**
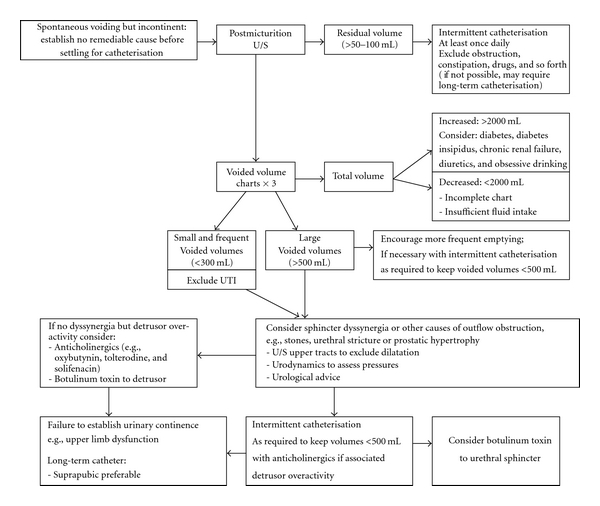
Managing urinary incontinence in patients with long term neurological conditions including primary progressive MS. (Printed with permission: RCP 2008 [25].)

Detrusor overactivity can be treated with anticholinergic medications (oxybutynin, imipramine, solifenacin, and tolterodine) and in severe cases with intravesical oxybutynin or botulinum toxin. Additional techniques for managing bladder include scheduled fluid intake, prompted voiding, avoidance of alcohol and caffeine, pelvic floor exercises and other behaviour modifications. For detrusor sphincter dysynergia regular attempts to void (light tapping), trial of antispasticity agents (baclofen), alpha adrenergic blocking agents (prazosin and clonidine), and anticholinergic medications (oxybutynin) with intermittent catheterization can be trialed. Detrusor underactivity causes incomplete bladder emptying and can be managed with intermittent catheterization; if these are unsuccessful, an indwelling catheter may be needed. Suprapubic catheterisation is preferable than urethral catheterization, as they have fewer complications including urinary infections, are easier to change, and permit normal sexual functioning. Figure 4 provides a flow chart for managing urinary incontinence in patients with long-term neurological conditions including PPMS [39]. 

Symptomatic lower urinary tract infections should be treated on the basis of a positive urine culture. Acidifying agents such as cranberry can reduce risk of recurrent urinary infections in neurogenic bladders. Symptomatic management of lower urinary tract symptoms includes the antidiuretic hormone desmopressin (DDAVP) nasal spray for nocturia and oral cannabinoids. Bladder retraining and pelvic floor exercises may be useful if patients are appropriately educated in conjunction with a physiotherapist. Patients should be provided with tips to prevent recurrent urinary infections such as awareness of signs of infection (cloudy urine or pain and odor), adequate fluid intake (8 glasses per day), increase urine acidity (vitamin C) or cranberry capsule daily, try to achieving complete bladder emptying, wiping front to back after going to the bathroom, and regular change of indwelling catheters (4–6 weekly).

**Figure 5:**
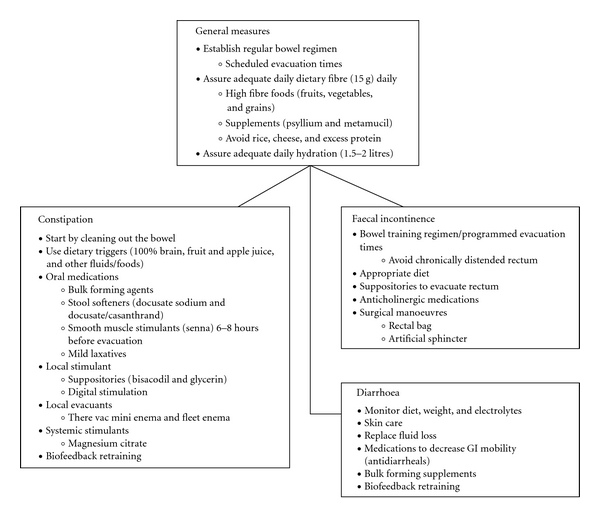
Management of bowel problems in MS. (Adapted from: Miller et al. [49].)

Bowel dysfunction has been reported in 50% of pwMS, with constipation, and faecal incontinence. These result from autonomic dysfunction and abnormal rectal function. Absent rectal sensations increase risk of faecal incontinence, which can reflect decreased rectal filling sensation, poor sphincter and pelvic floor contraction and decreased rectal compliance. A recent study identified female sex, higher disability, and urinary dysfunction as independent predictors of developing anorectal dysfunction [56]. Bowel programs need to be effective (i.e., complete within 60 minutes from beginning of program to bowel evacuation). Patient education includes review of diet and bowel habits. The optimization of consistency of bowel contents is ensured by adequate oral intake, a diet high in fibre and laxatives (bowel softeners such as coloxyl) if necessary. Next is the facilitation of the movement of the bowel contents, a combination of osmotic (e.g., lactulose) and stimulants (e.g., senna) is effective and is the mainstay medical treatment. The iso-osmotic laxative polyethylene glycol (Movicol) has been shown to be effective in chronic constipation and is used in resistant cases. Frequent use of enemas should be avoided. The timing of a bowel program ideally should be postprandial when the gut is most active. Rectal stretching (suppository) can facilitate the defecation reflex and assist bowel evacuation. A flow chart outlining bowel management in pwMS is shown in Figure 5. 

Sexual dysfunction in MS has been widely reported especially in patients with urinary symptoms [57]. Causes of sexual dysfunction may be primary (lack of lubrication, diminished genital sensations, erectile dysfunction), secondary (spasticity, pain, catheter care) or tertiary (marital difficulty, fear, and lack of confidence and self-worth). Men commonly report diminished libido, erectile and ejaculatory dysfunction. Women report diminished genital sensation, lubrication, and difficulty achieving orgasm. Rehabilitation includes education about intimacy and sexuality, management of fatigue, positioning, and mechanics, information about aids (tumescence devices), specific suggestions and techniques, and referral for sexual counselling. The use of oral phosphodiesterase inhibitors (e.g., sildenafil) have been successful in treating erectile dysfunction in men; their role in women has not been established. In addition to intracorporeal pharmacotherapy, papaverine has now been replaced by prostaglandin E1 by intracavernosal injection or urethral application.

#### 5.1.3. Mobility Related Symptoms

Mobility can be affected in MS from a combination of motor (weakness and spasticity), sensory (proprioception loss, ataxia), fatigue, and visual impairments.

**Spasticity**

Spasticity, a velocity-dependent increase in muscle tone, is a common complication of MS. Muscle shortening and restricted movements lead to decreased tissue compliance and biomechanical difficulties (contractures) which can limit a person's activity (mobility, ability to transfer, perform self-care tasks, and pain) and participation (unable to drive or work). Management of spasticity in pwMS is complicated due to lesions in the brain and spinal cord, numerous other secondary MS-related impairments, and their associated polypharmacy. There are limited studies which do not suggest improved outcomes of one strategy for managing spasticity compared with another. Two useful measure of spasticity include the spasm frequency scale [58] and the modified ashworth scale [59]. The spasm frequency scale is obtained from history, and is a 0–4 non interval scale: 0: no spasms, 4: 10 spontaneous spasms per hour. The modified ashworth scale is obtained after clinical examination and is also a non interval scale of 0–4 (although it includes a value for 1+), with 0: “no increase in tone”, 4: “affected part is rigid”. More recently, a systematic review found the Tardieu scale to be a more sensitive measure for spasticity; however, further validity validation of this scale for various muscle groups is required [60].

The treatment goals change with progression of disease. Early in the course of the disease, spasticity can interfere with functional activities and also cause gait inefficiency, which in turn increases fatigue. On the other hand, as muscles weaken, some patients rely on their spasticity to keep them on their feet. Carefully targeted intervention for those elements of spasticity that are unwanted can assist with energy conservation and so keep pwMS mobile and independent for longer. Later on, the focus of treatment is more on improving ease of maintaining hygiene, prevention of contractures and pain reduction. Management involves patient education, therapy intervention, and medication [28]. The aims of therapy are awareness of symptoms related to spasticity and awareness of factors that can worsen spasticity for example, noxious stimuli, sudden movements, anxiety; correct positioning and alignment, and a stretching program. The mainstay of treatment is maintaining muscle length, so the importance of positioning and physical management (such as a regular standing regimen) cannot be over-estimated. Drugs are an adjunct to these interventions and may be given orally or by intramuscular, intraneural, or intrathecal injection.

Oral antispasticity agents are first-line therapy for generalised spasticity [28]. The most commonly prescribed oral agents include baclofen, tizanidine, clonazepam, and dantrolene. Baclofen (gamma amino butyric acid agonist) remains the agent of first choice though its use is restricted by side effects including weakness, fatigue, and cognitive impairment. Tizanidine (central acting *α*2 adrenergic agonist) can be used in conjunction with baclofen or in isolation in patients who cannot tolerate or have no response to baclofen. Clonazepam (benzodiazepine) is particularly effective at treating nocturnal spasms. Its side effect profile includes sedation and effects on cognition. Dantrolene acts at the level of the muscle and can be used with any of the above agents for severe generalized spasticity. Its use is limited by its side-effect profile and poor tolerability. 4-Aminopyridine (Dalfampridine and Fampridine) (voltage-gated potassium channel blocker) has been shown to provide improvement in lower limb function, but toxicity with seizures and encephalopathy can occur at therapeutic doses [61, 62]. Other pharmacological agents such as Memantine did not show any benefit in treatment of spasticity [63]. Cannabis extracts are reported to have positive effect on spasticity and can be prescribed orally or via nasal spray [28]. Cannabinoids act on CB1 receptors in the CNS to inhibit cyclic AMP and voltage-dependent calcium channels, causing antispastic effect.

Intrathecal Baclofen (ITB) is effective for severe generalised spasticity, particularly in the lower limbs. It requires lower doses and has improved tolerability due to less sedative and cognitive side effects. However, ITB withdrawal syndrome (incorrect dosage and pump failure) can be life threatening, and therefore, this treatment should only be managed in specialist units [64]. For focal spasticity, botulinum toxin injected into the affected muscle(s) can be effective [65]. Other localized spasticity (adductor muscles) can be treated with phenol neurolysis. Surgical options (tendon release surgery) are reserved for severe spasticity, causing pain, interfering with care, and/or limiting activities of daily living.

**Ataxia**

Cerebellar problems such as tremor, ataxia, and incoordination are common in pwMS. The action (postural- intention) tremor reflects brainstem-cerebellar circuitary lesions and can be disabling and is often difficult to treat. Symptomatic treatment for tremor includes identification of type of tremor, trigger factors and part of body involved. Use of assistive devices (braces and support) and evaluation for therapy may be helpful. Limited benefit from drug therapy using Ondansetron (for cerebellar tremor), propanolol, and combined therapy with lamotrigine and gabapentin have been reported. Surgical ablative and stimulation techniques (ventral intermediate thalamic nucleus) are currently being trialed.

Truncal ataxia can occur in up to 70% of pwMS, often accompanied by tremor. It has a significant effect on motor coordination (similar to weakness) and interferes with balance and mobility, increasing predisposition to falls and injury. Patient education and safety in daily living tasks is emphasized as rehabilitation strategies, which include improvement in posture and alignment, proximal stabilization (pectoral and pelvic girdle musculature), coordination exercises, and assistive devices such as the use of distal weights around the wrists to dampen tremor and the use of walking frames or elbow crutches enhance gait stability. Falls prevention strategies and environmental modification (installation of grab rails, nonskid floor mats) can be helpful. In one small RCT (*n* = 23) [66], pwMS were randomized to specific physiotherapy strategies such as (a) facilitation therapy (individualized, passive and active manual assistance, postural control, and component practice as in Bobath technique) and (b) task oriented therapy (nonindividualized, hands off acceptance of compensatory strategies and functional tasks such as stair climbing and treadmill walking). Although patients in both groups showed improvement in gait scores, balance tests and global mobility indices, those in the facilitation group had nonsignificant trends towards greater benefit in all categories. Medications for ataxia are similar to those used for treating tremors (isoniazid, clonazepam, propanolol, gabapentin, and Ondansetron). A recent systematic review found no evidence that medication or neurorehabilitation strategies provide sustained improvement in ataxia in pwMS [67]. Surgical interventions such as thalamotomy or thalamic stimulation in MS have produced limited success.

#### 5.1.4. Pain and Paroxysmal Symptoms

Pain can be acute or chronic. The underlying mechanisms of pain in MS are unclear and have been linked with the differentiation and disinhibition of central and pain pathways [68, 69] with CNS lesions causing hyperexcitability and with increased neuronal activity at the site of the lesion in the spinal cord [70]. Acute pain may be associated with active inflammatory process. Chronic pain may be due to the MS process itself or from complications that arise from it such as trigeminal neuralgia, spasms/spasticity, and musculoskeletal posture and gait-related problems [71]. 

In one recent Australian series (*n* = 94) 60% of patients reported chronic pain, of these 61% had dysesthetic pain and 70% had episodic increases in pain [72]. Chronic pain in MS impacts on activities of daily living [71] and interferes with ability to work [73]. A recently published study (*n* = 62) performed cluster analysis to classify patients into three cognitive behavioral groups (adaptive copers, dysfunctional and interpersonally distressed) and suggested possible cut points to aid clinicians in classifying patients into clusters for individualized treatment [74]. The severity of depression is reported to be higher in persons with MS with chronic pain than those without pain. There is also increased interference with daily activities, more severe symptoms of depression and negative effect on relationships with partners and family [71]. Treatment of chronic pain has been discussed elsewhere [75]. A multidisciplinary team approach may be needed and referral to pain clinic may be helpful. 

A systematic approach initially using monotherapy to maximum doses before polytherapy is imperative Amitriptyline is effective for chronic dysesthetic pain. Carbamazepine is the drug of choice for trigeminal neuralgia if not tolerated then alternatives include gabapentin, lamotrigine, and phenytoin [76]. Transcutaneous electrical nerve stimulation to lower back of pwMS appears promising [77]. Surgical options are percutaneous procedures and rarely microvascular surgery [78]. Carbamazepine and gabapentin are agents of choice for other paroxysmal symptoms (tonic spasms, ataxia, or sensory symptoms like Lhermitte's). Cannabis-based preparations are effective for pain in pwMS [79] but are reserved for cases where standard therapies have failed or are not tolerated. There is no evidence to support routine use of intrathecal morphine for pain management in the MS population. Pregabalin, an isomer of GABA with selectively binds to the alfa2-delta protein of the voltage-gated calcium channels, has been shown to be efficacious in the management of peripheral neuropathic pain of various causes including MS [80, 81].

#### 5.1.5. Cognitive Deficits

Current estimates of prevalence of neuropsychological problems in MS are approximately 50% [82, 83]. The neurocognitive and behavioural deficits in MS, and suggested treatments are discussed in a recent review [84]. Cognitive problems result from affected pathways in the cerebral white matter (limbic system, the midbrain, and brainstem), which transmit to, and communicate with, higher-level cortical regions throughout the brain. These deficits can be a major impediment to rehabilitation and include: inability to store and to retrieve information, decreased memory, attention and speed of processing, and limitations in emotion, personality, and behaviour [84–86]. 

Many guidelines exist for neuropsychological research in MS [84]. Neuropsychological interventions are designed to enhance a person's ability to function in all areas of family and community life, which are meaningful for pwMS. A neuropsychological assessment can be helpful to delineate problems and suggest compensatory techniques. These include functionally oriented therapies based on specific deficits: compensatory strategies (using intact skills or external aids to improve function), substitution (learnt use of intact cognitive abilities to circumvent a problem), or scheduling (templates and structured programs) may assist with everyday living tasks.

A systematic review reported that cognitive behaviour therapies (CBT) were beneficial for pwMS in terms of coping with, and adjustment to MS [33]. Other specific cognitive rehabilitation protocols are being evaluated [87]. Although the evidence for individual interventions is limited, computer-based retraining program was shown to improve deficits related to attention [88]. Medications such as amantadine, glatiramer acetate, memantine, and donepezil failed to improve cognitive function in MS [89–92]; others such as methylphenidate have not yet been studied in pwMS.

#### 5.1.6. Visual and Brainstem Symptoms

Visual disturbances were reported by 58% of pwMS in one large cohort [93]. Referral to “low vision clinic” may be required for decreased visual acuity (optic neuritis). The visual dysfunction may also result from involuntary eye movement disorders (nystagmus and opsoclonus) [28]. Patient education, use of adaptive visual aids (prisms and magnifying lens), and occasionally medications such as baclofen, isoniazid, and gabapentin may be helpful for involuntary eye movements [94]. Referral to occupational therapy and low vision clinics can be helpful for neuromobility services such as practicing outdoor mobility to improve safe community access.

Vestibular involvement in MS is frequent and causes vertigo and often associated with other signs of brainstem dysfunction. Specific vestibular physiotherapy exercises (such as Cawthorne-Cooksey protocol) may be helpful. Effective speech therapy for dysarthria for MS includes control of speech rate, voice emphasis and power, and reduction in phrase length [95–97]. Dysphagia occurs in about 34%–43% of pwMS [98, 99]. Fatigue, tremors, weakness, and incoordination exacerbate dysphagia and dysarthria. Spasticity is worsened by malnutrition. For the most severely affected pwMS, maintenance of nutritional balance may require placement of a percutaneous peg for feeding. This requires specialized nutritional and speech pathology services. Videofluoroscopy and clinical assessment is recommended for more disabled persons [100]. Speech therapy can provide compensatory strategies to avoid aspiration, correct posture (sitting up when eating), alter food consistency, and provide education to prevent complications (pneumonia) [99].

#### 5.1.7. Psychiatric and Psychological Dysfunction

The prevalence of major depressive disorder in pwMS is reportedly between 27%–54%, and nearly double the prevalence in persons without MS over 12 months (15.7% versus 7.4%) [101]. There was also an age effect, with a prevalence of 25% in adults between 18 and 45 years. The relationship between depression and cognitive dysfunction, and treatment are discussed elsewhere [71]. Depression impacts' on all aspects of life and can amplify symptoms, leading to further limitation in function, and interferes with disease management [102]. Major depressive disorder is linked to objective cognitive difficulties (attention and memory) [103]. 

Selective serotonin reuptake inhibitors are widely used to treat depression in rehabilitation. One study (*n* = 630) compared CBT, the antidepressant Sertraline, and group psychotherapy [104]. CBT and Sertraline were more efficacious than group therapy, and improvement in depressive symptoms persisted at 6-month followup. Symptoms of depression also improved in persons who received an alternate approach—an eight-week telephone cognitive behavioural intervention compared to usual care [105]. This approach was adapted to address barriers such as transportation and access pwMS. Exercise improves mood, fatigue and QoL [105, 106] and is as effective as standard antidepressant medication and psychotherapy [107]. 

Other approaches to treat depression include behaviour activation (which treats depression by increasing access to positive reinforcement and decreasing frequency and intensity of aversive events and consequences) [108] and interpersonal therapy—an evidence based approach that focus' on role disputes and role transitions as a framework for therapy [109]. 

Psychosocial issues include inability to cope (patient and family), stress, financial considerations, and marital discord. A recent Australian study outlined factors impacting on MS caregivers in a community setting [110]. More strain was reported in caregivers caring for pwMS with depression, anxiety, and stress levels, with a poorer QoL for both the carer and care recipient. Education and support, stress management, and coping skills can positively influence health and wellbeing and may require clinical psychology and psychiatry. Neuropsychological counselling improved insight and social skills training compared with standard counselling, in reducing disinhibition and socially aggressive behaviour especially in cognitively impaired pwMS [111].

#### 5.1.8. General Reconditioning and Ambulation

Reduced physical activity and exercise due to MS limitations have been discussed elsewhere [112]. Causal factors include decreased muscle strength, aerobic capacity, maximal vital capacity, and increase in neuromuscular tension, fatigue, anxiety, and depression. Exercise programs do not alter the MS disease course but do prevent secondary effects of inactivity and improve fatigue and sense of well-being. An integrated exercise program incorporates: a daily passive range of motion, an active range of motion with gravity eliminated or against gravity as allowed by strength, and specific muscle training (three sets of 10 repetitions) is recommended for focal weakness, when fatigue and heat sensitivity are issues [112, 113]. Active exercise for 20–30 minutes 3 times per week, with a 5 minute warmup and cool-down, stretching for lower limbs and back is effective [112], while aerobic exercises for cardiovascular fitness are important for overweight persons [114]. 

Gait is impaired by weakness, spasticity, incoordination, balance, fatigue, and visual disturbances. For ambulation, a graded program should improve trunk control and balance, followed by normalizing tone, flexibility, and range of motion and then strength. Graded sitting and standing tolerance program and tilt table routine prior to gait training may be required. Proprioceptive, tactile, and visual cues are also helpful. Specific ambulation aids (elbow crutches, walking frames, and ankle foot orthoses) and mobility devices (wheel chairs and scooters) can decrease energy expenditure and improve safety and endurance [115]. A person's strength, motor control, cognition, and emotional response are all considered prior to prescription. Wheelchairs are customized for each person, such as appropriate seating, posture support, tilt in space mechanism, and manipulation of components (arm rests, foot plates). Scooters assist those with ataxia and fatigue. Weighted wrist cuffs and walkers may help dampen tremors [116].

#### 5.1.9. Activities of Daily Living

Improvement in functional independence and maintenance is a key rehabilitation goal. Principles of occupational therapy (OT) in MS have been previously discussed [117]. OT was effective in improving function in pwMS, using retraining techniques for personal, domestic, and community tasks, mainly in inpatient settings [34]. However, in a recent systematic review [31] patient education and energy conservation strategies in MS were found to be inconclusive due to methodological weakness of included studies. OT should concentrate on activities that pwMS would use in practice, rather than on activities that people may not value because of environmental or behavioural circumstances [85].

#### 5.1.10. Driving

Although a recent study did not find excessive risk for fatal road accidents in pwMS [118], many issues impact on driving, especially cognitive and perceptual considerations [119]. Driving assessments may be required based on each individual's deficits. In persons with PPMS, if there are concerns, then a driving assessment by the occupational therapist is recommended. Fatigue-related issues due to MS may impact on the ability of the pwMS to drive for 45 minutes without a break—this has implications for holding a full driving license (Australia). Restrictions such as driving during day time only (poor night vision) or driving in localized area may be required. For more severely affected individuals, other specialized driving adaptations such as hand brakes, use of spinner knob on driving wheel, extra rear view mirrors, and motorized pulleys for folding and storage of wheelchairs may be needed.

#### 5.1.11. Employment

An estimated 65% of pwMS were working at the time of their diagnosis, and between 25% and 35% of these persons remain in work force 5–10 years of diagnosis [120]. Fatigue, urinary urgency and incontinence, and visual and mobility issues are the main barriers for continued employment. Many pwMS leave workforce prematurely, or on advice of a well-meaning health care provider or family member. Rehabilitation input may assist in continued employment. Reasonable accommodations for MS include flexible working hours, work at home options, transportation, accessible work environment (bathroom, desk), memory aids (planners and diaries), vision aids (voice recognition software), and air-conditioning. Return-to-work programs are customized, graded (gradual increase in working hours), or altered to suit the individual with MS [121]. These programs are coordinated by the Vocational Rehabilitation Services, in collaboration with the employee, employer and the treating rehabilitation team. Vocational rehabilitation interventions for pwMS focus on job retention strategies rather than retraining for new jobs. There are a very limited number of high-quality studies at present that address the efficacy of vocational rehabilitation in the MS population. A recent review, therefore, found insufficient evidence as yet to support vocational rehabilitation in pwMS [122].

## 6. Summary

The multiple concurrent MS-related physical, cognitive, emotional, and social issues make rehabilitation challenging in persons with PPMS. Rehabilitation measures do not alter the course of MS disease. The overriding principle in setting goals for a pwMS is to maximize functional independence and safety, minimize complications and problems that result from decreased mobility, compensate for loss of function, and improve quality of life. With disease progression a “neuropalliative” approach is required. Rehabilitation should be viewed as an ongoing process to anticipate problems and to maintain and restore maximum function and QoL for persons with PPMS.

## Figures and Tables

**Box 1:**
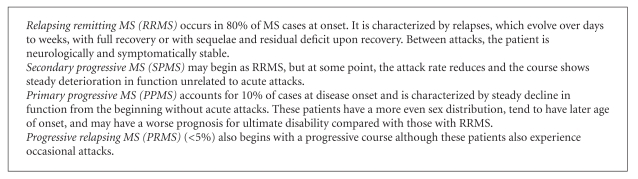
Types of MS. Adapted from Polman et al. [11].

**Box 2:**
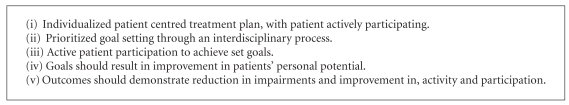
Subcomponents of comprehensive rehabilitation. Adapted from: Steins et al. [16].

**Box 3:**
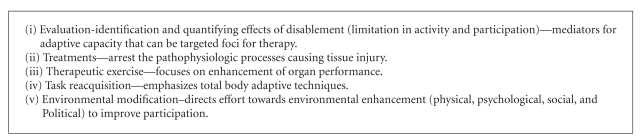
Phases in rehabilitation process. Adapted from: Steins et al. [16].

**Box 4:**
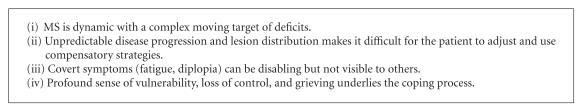
Challenges in MS rehabilitation.

**Box 5:**
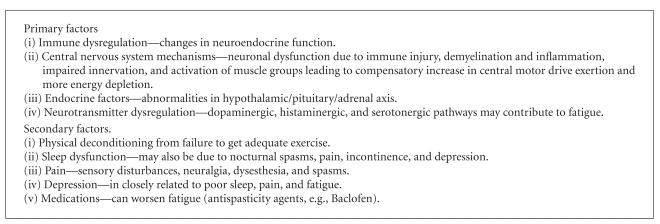
Primary and secondary factors in multiple sclerosis fatigue.  (Adapted from: MacAllister and Krupp [48]).

**Table 1:** McDonald criteria for MS.

Clinical (Attacks)	Objective lesions	Additional requirements to make a diagnosis
Two or more	Two or more, or objective clinical evidence of one lesion with reasonable historical evidence of a prior attack	None

Two or more	One	Dissemination in space, demonstrated by: one or more T2 lesion in at least 2 of 4 MS-typical regions of the CNS (periventricular, juxtacortical, infratentorial, or spinal cord) or await a further clinical attack implicating a different CNS site

One	Two or more	Dissemination in time, demonstrated by: simultaneous presence of asymptomatic gadolinium-enhancing and nonenhancing lesions at any time; or A new T2 and/or gadolinium-enhancing lesion(s) on follow-up MRI, irrespective of its timing with reference to a baseline scan or await a second clinical attack

One (clinically isolated syndrome)	One	Dissemination in space and time, demonstrated by: for DIS: one or more T2 lesion in at least 2 of 4 MS-typical regions of the CNS (periventricular, juxtacortical, infratentorial, or spinal cord) or await a second clinical attack implicating a different CNS site and For DIT: simultaneous presence of asymptomatic gadolinium-enhancing and nonenhancing lesions at any time or a new T2 and/or gadolinium-enhancing lesion(s) on follow-up MRI, irrespective of its timing with reference to a baseline scan or await a second clinical attack

Insidious neurological progression suggestive of MS (PPMS)		One year of disease progression (retrospectively or prospectively determined) plus 2 of 3 of the following criteria: (1) evidence for DIS in the brain based on one or more T2 lesions in the MS-characteristic (periventricular, juxtacortical, or infratentorial) regions, (2) evidence for DIS in the spinal cord based on tow or more T2 lesions in the cord (3) positive CSF (isoelectric focusing evidence of oligoclonal bands and/or elevated IgG index)

MS: multiple sclerosis; CNS: central nervous system; MRI: magnetic resonance imaging; DIS: dissemination in space; DIT: dissemination in time; PPMS: primary progressive multiple sclerosis; CSF: cerebrospinal fluid; IgG: immunoglobulin G. (Adapted from Polman et al. [12].)

**Table 2:** International classification of functioning, disability, and health (ICF) categories for the components: body “function” and body “structure” included in the “core” set for persons with MS for comprehensive multidisciplinary assessments.

ICF code	ICF category title
*Body function*	
B122	global psychosocial functions
B130	energy and drive functions
B144	memory function
B147	psychomotor function
B126	temperament and personality functions
B152	emotional functions
B164	higher level cognitive functions
B140	attention function
B160	thought functions
B114	orientation functions
B210	seeing function
B265	touch function
B260	proprioceptive function
B280	sensation of pain
B235	vestibular functions
B320	articulation functions
B330	fluency and rhythm of speech functions
B310	voice functions
B455	exercise tolerance functions
B435	immunological system functions
B525	defecation function
B640	sexual functions
B620	urination functions
B760	control of voluntary movement functions
B750	motor reflex functions
B730	muscle power functions
B735	muscle tone functions
B755	involuntary movement reaction functions
B770	gait pattern functions
B710	mobility of joint functions
B780	sensations related to muscle and movement functions
B715	stability of joint functions
*Body structure*	
S110	structure of brain
S120	structures spinal cord and related
S610	structure of urinary system
S720d	structure of shoulder region
S730	structure of upper extremity
S750	structure of lower extremity
S760	structure of trunk

(Adapted from Khan and Pallant [18].)

**Table 3:** International classification of functioning, disability and health (ICF) categories for the components: “activities and participation” and “environmental factors” included in the “core” set for persons with MS for comprehensive multidisciplinary assessments.

ICF code	ICF category title
*Activities and participation*	
D160	focusing attention
D172	calculating
D175	solving problems
D220	undertaking multiple tasks
D240	handling stress and other psychological demands
D163	thinking
D166	reading
D177	making decisions
D230	carrying out daily routine
D210	undertaking a single task
D330	speaking
D350	conversation
D355	discussion
D345	writing messages
D315	communicating with-receiving-nonverbal messages
D310	communicating with-receiving-spoken messages
D360	using communication devices and techniques
D335	producing nonverbal messages
D325	communicating with-receiving-written messages
D415	maintaining a body position
D420	transferring oneself
D445	hand and arm use
D450	walking
D455	moving around
D410	changing basic body position
D430	lifting and carrying objects
D440	fine hand use
D460	moving around in different locations
D456	moving around using equipment
D470	using transportation
D475	driving
D510	washing oneself
D530	toileting
D540	dressing
D550	eating
D520	caring for body parts
D560	drinking
D570	looking after one's health
D630	preparing meals
D640	doing housework
D650	caring for household objects
D620	acquisition of goods and services
D770	intimate relationships
D710	basic interpersonal interactions
D720	complex interpersonal interactions
D740	formal relationships
D760	family relationships
D750	informal social relationships
D850	remunerative employment
D845	acquiring keeping and terminating a job
D870	economic self sufficiency
D825	vocational training
D855	nonremunerative employment
D860	basic economic transactions
D920	recreation and leisure
D910	community life
D930	religion and spirituality
*Environmental Factors*	
E120	products or technology for personal indoor/outdoor mobility and transportation
E135	products and technology for employment
E115	products or technology for personal use in daily living
E125	products and technology for communication
E130	products and technology for education
E155	design, construction, building products, and technology of buildings for private use
E150	design, construction, building products, and technology of buildings for public use
E110	products or substances for personal consumption
E165	assets
E225	climate
E210	physical geography
E310	immediate family
E320	friends
E355	health professionals
E340	personal care providers and personal assistants
E360	health related professionals
E325	acquaintances, peers colleagues, neighbours, and community members
E330	people in positions of authority
E315	extended family
E410	individual attitudes of immediate family members
E420	individual attitudes of friends
E450	individual attitudes of health professionals
E455	individual attitudes of health related professionals
E425	individual attitudes of acquaintances, peers colleagues, neighbours, and community members
E440	individual attitudes of personal care providers and personal assistants
E460	societal attitudes
E430	individual attitudes of people in positions of authority
E570	social security services, systems, and policies
E575	general social support services, systems, and policies
E525	housing services, systems, and policies
E540	transportation services, systems, and policies
E590	labour and employment services, systems, and policies
E530	utilities services, systems, and policies
E510	services, systems and policies for the production of consumer goods
E515	architecture and construction services, systems, and policies
E580	health services, systems, and policies

(Adapted from Khan and Pallant [18].)

**Table 4:** Key skills in neurological palliative care and rehabilitation.

*Every physician should have an understanding of the general principles of management and should also be aware of when and where to refer*	
*if more specialist advice is needed in the areas shown below*	

Exposure to people with long-term neurological conditions	(i) Understanding disease progression and prognosis

Symptom control	(i) Ability to control key symptoms including: (1) pain in neurological conditions (2) breathlessness (3) nausea/vomiting (4) anxiety/depression (5) spasticity management (6) 24 hour postural support (7) Bladder and bowels (8) Seizure control

Communication	(i) Basic understanding of common communication problems including dysphasia, dysarthria, cognitive speech disorders, and the different approaches to their management. (ii) Ability to communicate with people who have cognitive/communication impairments (1) using assistive communication devices (iii) Communicating with patient and family (1) breaking bad news (2) addressing end of life decisions and advance care planning which will include choice over place of care. (3) Managing expectations.

Legal issues	(i) Ability to assess for mental capacity and to assist people to make advance decisions and statements. (ii) Understanding of the Mental Capacity Act 2005 and ability to work alongside lasting power of attorney/court appointed deputy or independent mental capacity advocates.

*Additional skills for physicians specializing in neurological palliative care and rehabilitation *	

Specialist interventions	(i) Local and intrathecal interventions for spasticity (e.g., injection of botulinum toxin/phenol and use of baclofen pumps). (ii) Specialist procedures for pain control. (iii) Management of confusion/unwanted behaviours–management under sections of the Mental Health Act 1983 (iv) Ventilation

Specialist equipment	(i) Wheelchair seating systems (ii) Environmental control systems (iii) Specialist communication aids

Counselling and psychological support	(i) Dealing with loss and fear of loss (ii) Spiritual support (iii) Bereavement–past and future

Welfare advice	(i) Understanding the social care system and benefits (ii) Vocational support

Additional sources of help and support	(i) Understanding the interaction between health, social services and voluntary support agencies (ii) Negotiating skills in obtaining services

(Printed with permission: RCP 2008 [25].)

**Table 5:** Frequency of limitations reported by persons with MS (*N* = 101) linked with ICF categories for the components: body function, body structure, activity and participation, and environmental factors (those reported by at least one third of MS patients are listed below).

ICF code	Chapter title	ICF code description	Total number of participants linked responses. *n*, %	Number of participant and stage of disease			
				RR	SP	PP	rr-SP
		*Body function*					

b130	Global mental functions	Energy and drive functions	98, 97.03	51	26	14	7
b134		Sleep	84, 83.17	47	21	11	5
b140	Specific mental functions	Attention	66, 65.35	37	17	9	3
b144		Memory	62, 61.39	37	16	4	5
b152		Emotional functions	97, 96.04	50	26	14	7
b210	Seeing and related functions	Seeing	47, 46.53	24	16	4	3
b235	Hearing vestibular	Vestibular (incl. balance functions)	71, 70.30	34	19	13	5
b265	Sensory functions	Touch	34, 33.66	15	10	7	2
b280	Pain	Sensation of pain	76, 75.25	39	19	12	6
B455	CVS and respiratory functions	Exercise tolerance functions*	97, 96.04	50	27	13	7
b525	Digestive system	Defecation	89, 88.12	49	21	14	5
b620	Urinary functions	Urination functions	94, 93.07	50	24	13	7
b640	Genital and reproductive	Sexual functions	57, 56.44	32	15	7	3
b730	Muscle functions	Muscle power	96, 95.05	50	27	13	6
b735		Muscle tone	94, 93.07	50	26	13	5
b740		Muscle endurance function*	93, 92.08	49	25	12	7
b760	Movement functions	Control of voluntary movement functions*	66, 65.35	37	18	8	3
b770		Gait pattern functions*	99, 98.02	51	27	13	8

		*Body Structure*					

s110	Nervous system	Brain	100, 99.01	50	28	14	8
s610	Genitourinary system	Urinary system	93, 92.08	49	25	12	7
s730	Structures related to movement	Upper extremity (arm, hand)	44, 43.56	25	10	7	2
s750		Lower extremity (leg, foot)	97, 96.04	49	27	14	7
s760		Trunk	85, 84.16	44	23	12	6

		*Activities and participation*					

d160	Applying knowledge	Focussing attention	70, 69.31	39	16	9	6
d175		Solving problems	34, 33.66	22	8	2	2
d177		Making decisions	59, 58.42	35	16	5	3
d220	General tasks and demands	Undertaking multiple tasks	88, 87.13	47	24	12	5
d230		Carrying out daily routine	80, 79.21	48	17	10	5
d240		Handling stress and other psychological demands	101, 100.00	51	28	14	8
d430	Mobility	Lifting and carrying objects	53, 52.48	30	12	8	3
d440		Fine hand use (picking up, grasping)	51, 50.50	26	13	9	3
d445		Hand and arm use	37, 36.63	21	9	4	3
d450		Walking	101, 100.00	51	28	14	8
d455		Moving around*	99, 98.02	51	27	14	7
d465		Moving around and using equipment (wheelchair, skates, etc.)	98, 97.03	50	27	14	7
d470		Using transportation (car, bus, train, plane, etc.)	100, 99.01	51	27	14	8
d475		Driving (riding bicycle and motorbike, driving car etc.)	99, 98.02	51	27	14	7
d510	Self care	Washing oneself (bathing, drying, washing hands, etc.)	41, 40.59	26	9	4	2
d520		Caring for body parts (brushing teeth, shaving, grooming, etc.)	40, 39.60	24	8	5	3
d570		Looking after one's health	88, 87.13	47	23	14	4
d620	Domestic life	Acquisition of goods and services (shopping, etc.)	92, 91.09	50	24	12	6
d630		Preparation of meals (cooking etc.)	89, 88.12	48	24	12	5
d640		Doing housework (cleaning washing, laundry, and ironing)	94, 93.07	51	23	14	6
d650		Caring for household objects*	84, 83.17	46	22	12	4
d660		Assisting others	87, 86.14	48	22	13	4
d750	Interpersonal relationships	Informal social relationships	35, 34.65	19	12	2	2
d760		Family relationships	73, 72.28	42	16	11	4
d770		Intimate relationships	61, 60.40	35	15	7	4
d845	Work	Acquiring keeping and terminating a job*	73, 72.28	39	19	11	4
d850		Remunerative employment	90, 89.11	45	24	13	8
d870	Economic life	Economic self-sufficiency	84, 83.17	44	22	13	5
d910	Community life	Community Life	79, 78.22	40	21	13	5
d920		Recreation and leisure	97, 96.04	50	26	14	7

		*Environmental Factors*					

e110	Products and technology	For personal consumption (food, medicines)	101, 100.00	51	28	14	8
e120		For personal indoor and outdoor mobility and transportation	91, 90.10	47	25	12	7
e150		Design, construction and building products and technology of buildings for public use	70, 69.31	39	18	9	4
e210	Natural environment	Physical geography*	39, 38.61	21	11	5	2
e225		Climate	99, 98.02	50	28	14	7
e310	Support and relationships	Immediate family	45, 44.55	27	9	7	2
e315		Extended family*	42, 41.58	26	9	3	4
e460	Attitudes	Societal attitudes	31, 30.69	13	9	5	4
e540	Services, systems, and policies	Transportation services, systems and policies	68, 67.33	38	17	8	5
e580		Health services, systems and policies	79, 78.22	45	18	11	5

(Adapted from Khan and Pallant [19].)

**Table 6:** Measures of multiple sclerosis related fatigue.

Name of scale	Author, year [ref.]	Population	Specified fatigue subscales	No. of items	Scoring
Modified fatigue impact scale	Paralysed Veterans of America 1998 [42]	MS	Physical, cognitive and psychosocial	21	Likert scale
Rochester fatigue diary	Schwid et al., 2002 [43]	MS	Lassitude (reduced energy)	12 (1 item, 12 times over 24 h)	Visual analogue scale
Fatigue descriptive scale	Iriarte et al., 1999 [44]	MS	Spontaneous mention of fatigue, antecedent conditions, frequency, and impact on life	5	0–3
Fatigue impact scale	Fisk et al., 1994 [45]	MS	Physical, cognitive, and psychosocial	21	0–4
Fatigue assessment instrument	Schwartz et al., 1993 [46]	Lynne, Chronic fatigue syndrome, lupus, Ms, dysthymia, healthy	Fatigue severity, situation specificity, consequences of fatigue, and responds to rest/sleep	29	1–7
Single item visual analogue scale of fatigue	Krupp et al., 1989 [47]	MS, lupus, healthy	None	1	Visual analogue scale
Fatigue severity scale	Krupp et al., 1989 [47]	MS, lupus, healthy	None	9	Likert scale

(Adapted from: MacAllister and Krupp [48].)
